# A Novel, Integron-Regulated, Class C β-Lactamase

**DOI:** 10.3390/antibiotics9030123

**Published:** 2020-03-14

**Authors:** Maria-Elisabeth Böhm, Mohammad Razavi, Carl-Fredrik Flach, D. G. Joakim Larsson

**Affiliations:** 1 Centre for Antibiotic Resistance Research (CARe), University of Gothenburg, Gothenburg, Sweden; maria-elisabeth.bohm@gu.se (M.-E.B.); mohammad.razavi@gu.se (M.R.); carl-fredrik.flach@microbio.gu.se (C.-F.F.); 2 Department of Infectious Diseases, Institute of Biomedicine, Sahlgrenska Academy, University of Gothenburg, 405 30 Gothenburg, Sweden

**Keywords:** Class C β-lactamase, integron, functional metagenomics, antibiotic resistance

## Abstract

AmpC-type β-lactamases severely impair treatment of many bacterial infections, due to their broad spectrum (they hydrolyze virtually all β-lactams, except fourth-generation cephalosporins and carbapenems) and the increasing incidence of plasmid-mediated versions. The original chromosomal AmpCs are often tightly regulated, and their expression is induced in response to exposure to β-lactams. Regulation of mobile *ampC* expression is in many cases less controlled, giving rise to constitutively resistant strains with increased potential for development or acquisition of additional resistances. We present here the identification of two integron-encoded *ampC* genes, *bla*_IDC-1_ and *bla*_IDC-2_ (integron-derived cephalosporinase), with less than 85% amino acid sequence identity to any previously annotated AmpC. While their resistance pattern identifies them as class C β-lactamases, their low isoelectric point (pI) values make differentiation from other β-lactamases by isoelectric focusing impossible. To the best of our knowledge, this is the first evidence of an *ampC* gene cassette within a class 1 integron, providing a mobile context with profound potential for transfer and spread into clinics. It also allows bacteria to adapt expression levels, and thus reduce fitness costs, e.g., by cassette-reshuffling. Analyses of public metagenomes, including sewage metagenomes, show that the discovered *ampCs* are primarily found in Asian countries.

## 1. Introduction

AmpC-type β-lactamases, also called class C β-lactamases (Ambler structural classification) or class 1 cephalosporinases (functional classification), have gained clinical relevance due to their broad spectrum of activity against penicillins, cephalosporins, cephamycins, and monobactams, while not being inhibited by common β-lactamase inhibitors like clavulanic acid and tazobactam [1,2]. Major clinical concerns are the spread of plasmid-mediated AmpCs, the development of high-level resistance against a broad spectrum of β-lactams by mutations in the regulatory systems that control their expression, and the subsequent accumulation of resistance genes to several antibiotics [3]. 

After the introduction of later-generation cephalosporins, monobactams, and carbapenems in the 1980s to avoid resistance by known β-lactamases, overproduction of chromosomal AmpC was the first detected broad-spectrum resistance mechanism [3,4]. Nowadays, AmpCs are less prevalent than extended spectrum β-lactamases (ESBLs), but they have a similar substrate spectrum [2,5]. In addition, several AmpC β-lactamases cause resistance to carbapenems above the clinical threshold in a porin-deficient background [6,7,8]. This potential, in combination with the frequent occurrence of AmpCs in multidrug-resistant isolates, is rather worrisome [9,10,11]. 

AmpCs and other penicillin-binding proteins (not necessarily β-lactamases) are chromosomally encoded in both gram-positive and gram-negative bacteria [3]. Research on their expression and regulation has mostly focused on gram-negatives, where *ampC* expression normally is tightly regulated in response to β-lactam exposure and cell wall damage [3,12,13]. In many *Enterobacteriaceae* and in *P. aeruginosa*, the repressor AmpR, one or several versions of the regulatory enzyme AmpD, and the inner membrane oligopeptide permease AmpG control the inducible expression of *ampC* [14,15,16]. Different β-lactams induce *ampC* expression at different levels, and they are to varying degrees vulnerable to hydrolyzation. Penicillins and first-generation cephalosporins are strong inducers, and at the same time very good substrates for AmpCs. Consequently, many later-generation β-lactams are weak inducers and substrates [3,17]. In contrast, cefoxitin and imipenem are both strong inducers, leading to increased resistance against other β-lactams and inhibitors, but are poor substrates themselves (especially imipenem) [17,18]. Production of large quantities of AmpC achieves high-level resistance, even against the poorer substrates, such as cephalosporins and monobactams. 

Overexpression or de-repression of chromosomal AmpC is mainly caused by mutations in the genes *ampD* or *ampR*, or more rarely by mutations in *ampG* [3]. In *E. coli*, *A. baumannii*, and *Shigella*, expression is non-inducible, due to the lack of the *ampR* gene, and *ampC* expression is instead regulated by promoter and growth rate-dependent attenuation [15,19,20,21]. Mutations in the promoter or attenuator sequence can lead to increased expression [19,22,23]. In *Aeromonas*, *ampC* expression was first found to be controlled by the two-component regulatory system BlrAB (**β**-**l**actam **r**esistance) [24]. BlrAB-like systems were later shown to be global metabolic regulators—the homolog in *E. coli* and *P. aeruginosa* is the CreBC (**c**arbon source **re**sponsive) system—that affect cell wall recycling, biofilm formation, and general fitness as well [13,25]. 

Chromosomally encoded AmpCs have been known for decades in *Enterobacteriaceae*, before the first plasmid-borne AmpC was reported in 1989 [26]. Since then, plasmid-mediated AmpCs have spread quickly to various bacteria, and often resulted in multi-resistant pathogens [4,27]. Plasmid-encoded AmpCs are now common all over the world; they originate from the chromosomal versions, contain additional regulatory possibilities, and appear both inducible and in a perpetually de-repressed state [11,23]. They have been mobilized by different mechanisms, such as transposons and insertion sequence (IS) elements (either with their original expression regulation [28] or controlled by a promoter provided by the mobile element [29,30,31,32]), and are often associated with integrons [3,4,28,33,34,35]. These mobile elements, e.g., IS*26*, IS*Ecp1* or IS*CR1*, have inserted in close vicinity or within an integron, and form larger composite structures with other transposable elements. However, to the best of our knowledge, *ampC* genes have not yet been reported as a gene cassette in an integron. 

Integrons are genetic elements that acquire, shuffle, and express promoter-less gene cassettes to regulate the expression of a variety of accessory factors, including virulence, secondary metabolism, and antimicrobial or metal resistance genes [36]. Integrons consist of an integrase gene (*intI*) responsible for the specific excision and integration of gene cassettes at the *attI* recombination site. Gene cassettes are expressed from the constitutive promoter P_c_ embedded in *intI* and the region between *intI* and *attI*. The distance from this promoter determines the level of their expression [37]. All gene cassettes include *attC*, an imperfect inverted repeat, at the 3’ end, which is recognized as target recombination site by the integrase [38]. Class 1 integrons, developed from chromosomal structures, have been mobilized and spread rapidly as a consequence of natural selection and co-selection [39]. A large number of available resistance gene cassettes and the linkage to transposable elements have made this process possible, immensely accelerated by the human use of antibiotics and disinfectants [38,40,41]. The known pool of resistance gene cassettes is comprised of at least 130 different genes [42]. They include a variety of aminoglycoside modifying enzymes, chloramphenicol, quinolone, macrolide, fosfomycin, and trimethoprim resistances, as well as quarternary ammonium compound efflux proteins and β-lactamases. Some class A β-lactamases, like GES, KPC, and BEL, as well as the class D β-lactamase OXA are well known as resistance gene cassettes [43,44,45]. Class B metallo-β-lactamases, like IMP or VIM [46,47], are also abundant in class 1 integron cassettes, but to the best of our knowledge, no class C β-lactamase has been reported in integrons so far. 

If class C β-lactamases are indeed present as gene cassettes, they can take advantage of a successful transmission and expression system already harbored by a multitude of mobile elements and bacteria (both pathogenic and non-pathogenic). The ability of integrons to accumulate resistances also favors co-selection, and would enhance the spread of cassette-encoded *ampC*. To answer the question of whether *ampC* genes appear as gene cassettes, we searched all sequences encoding potential *ampC* genes from NCBI’s non-redundant protein database and the metagenomic datasets from a previous study, which have focused specifically on the identification of antibiotic resistance genes in class 1 integrons [48]. We discovered two novel AmpC proteins that are encoded as gene cassettes and verified their functionality in *E. coli*. Expression under control of a class 1 integron constitutes an additional way to escape tight regulation control and to facilitate fast spread. 

## 2. Results

### 2.1. A Novel Class C β-lactamase Occurring in Gene Cassettes

To determine the general abundance of *ampC* gene cassettes, we searched all complete and draft bacterial genomes in NCBI’s assembly database for the 311 known *ampC* genes from the comprehensive antibiotic resistance database (CARD) [49] with identity greater than 70% and coverage greater than 50%, as thresholds. We then checked the DNA sequences 2.5 kb up- and downstream of the 20,836 resulting open reading frames (ORFs) for integron attachment sites (*attC*) using IntegronFinder [50]. Only one *ampC* gene was detected, *bla*_CMY-2_ of *Salmonella enterica* (GenBank: AAGERX010000065.1), with *attC* sites in close vicinity. A more detailed analysis showed that *bla*_CMY-2_ is merely located adjacent to the last cassette of a class 1 integron. Furthermore, *bla*_CMY-2_ and the downstream region have appeared in several bacterial genomes without the integron, suggesting that the detected *bla*_CMY-2_ is not a gene cassette (Figure S1, Supplementary Materials). 

Since no known *ampC* gene was discovered as a gene cassette in sequenced isolates, we chose to search the functional metagenomics amplicons containing only integron gene cassettes that were selected on cefotaxime [48] for *ampC* genes with the same method. Preparation of the functional metagenomic amplicons is summarized briefly as follows: DNA was isolated from river sediments contaminated with municipal and hospital sewage. Gene cassettes were amplified from these samples, with primer pairs targeting the gene cassette array of class 1 integrons [48,51]. The amplification products were cloned into the vector pZE21-P*_bla_* and screened for resistance against different antibiotics in *E. coli* DH10β. The resistant clones were scraped off the selection plates and used as templates to prepare barcoded amplicons for long-read sequencing (PacBio). Any putative or known *ampC* recovered from the cefotaxime selection reads can be considered mobile and prone to occur in pathogens. This search resulted in 101 ORFs. After assuring that they were complete and flanked by attachment sites, 15 unique but still similar ORFs (at least 94% nucleotide identity) remained, and none of them was a known *ampC.* Twelve ORFs appeared each on a single PacBio read. They had sporadic mismatches and more than 99% nucleotide similarity to one of the other three ORFs, suggesting that the emergence of these variants was due to possible sequencing errors. Moreover, two of the three abundant ORFs differed consistently by just one nucleotide in all their identified reads. We chose to verify the functionality of one of these almost identical ORFs, as well as the other abundant ORF (the number of unique reads carrying them are listed in Table S1 in the Supplementary Materials). The two selected ORFs share 95% amino acid sequence identity between them, but are less than 85% identical to the closest related sequences that are currently available in the NCBI non-redundant protein database: WP_123086336.1 and WP_095207490.1. Consequently, we considered them a novel family of class C β-lactamases and named them *bla*_IDC-1_ and *bla*_IDC-2_ (integron-derived cephalosporinase). The two new AmpC variants were not recognized earlier by sequence homology-based analyses of the same metagenomic DNA samples using entire genes as templates [51,52,53], since the identity threshold was too stringent. 

**Figure 1:**
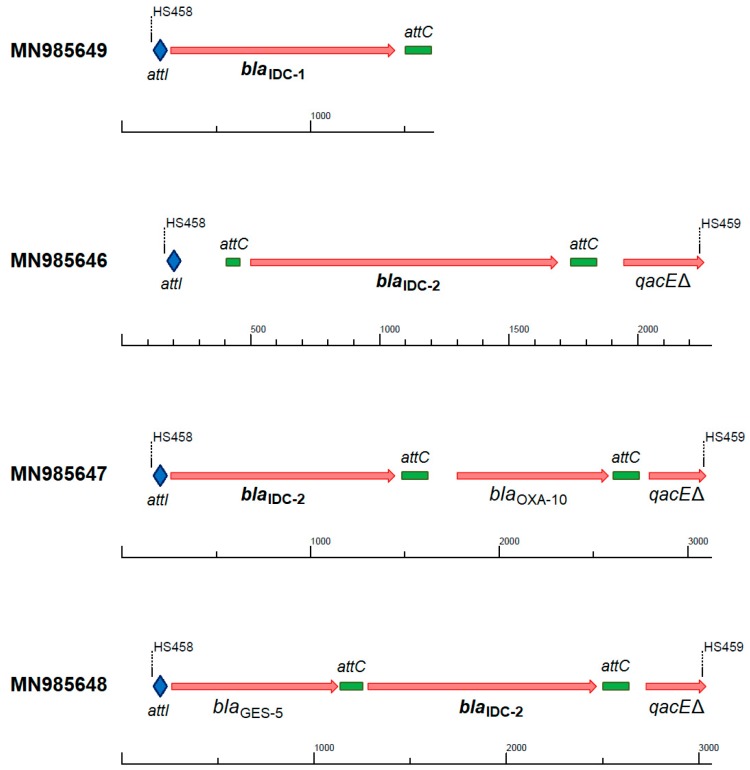
Genetic context and nucleotide accession numbers of the *bla*_IDC_ gene cassettes recovered from the amplified functional metagenomics dataset of class 1 integrons (primer pair HS458-HS459) [48,56]. Attachment sites: *attI* marked as blue rhomb, *attC* marked as green rectangle; *bla*_IDC-1_ and *bla*_IDC-2_: novel integron-derived **c**ephalosporinases; *bla*_OXA-10_: class D β-lactamase OXA-10; *bla*_GES-5_: class A β-lactamase GES-5 (carbapenemase); *qacE*Δ: quarternary ammonium compound resistance protein (truncated).

The *bla*_IDC-2_ gene cassette was identified in three different contexts in class 1 integrons, while *bla*_IDC-1_ always occurred as the first cassette in truncated reads (Figure 1). All three cassette arrays were also successfully identified by IntegronFinder from the amplicon reads. The *ampC* gene appeared as the first or second cassette, often combined with the class D β-lactamase OXA-10 [54] or the carbapenemase GES-5 [55], which can confer resistance against almost all β-lactam antibiotics to any strain harboring this integron. 

### 2.2. Evidence for Class C β-lactamase Activity: Resistance Profile

Functional screening of the metagenomic libraries included four β-lactam antibiotics (cefotaxime, ertapenem, imipenem, and meropenem [48]). The two identified novel *ampC* genes were both clearly selected on cefotaxime, but not on carbapenems (Table S1 in the Supplementary Materials), and were assumed to be AmpCs on the basis of sequence similarities. AmpCs have a broad substrate spectrum, but as their expression is variable in clinical isolates, and consequently the corresponding resistance levels, there is no standardized detection method [4,57,58]. We therefore chose to test β-lactamase activity with both the disk diffusion method and Etest for the determination of minimal inhibitory concentration (MIC) values against a selection of different cephalosporins, carbapenems, monobactams, and inhibitors. 

**Figure 2:**
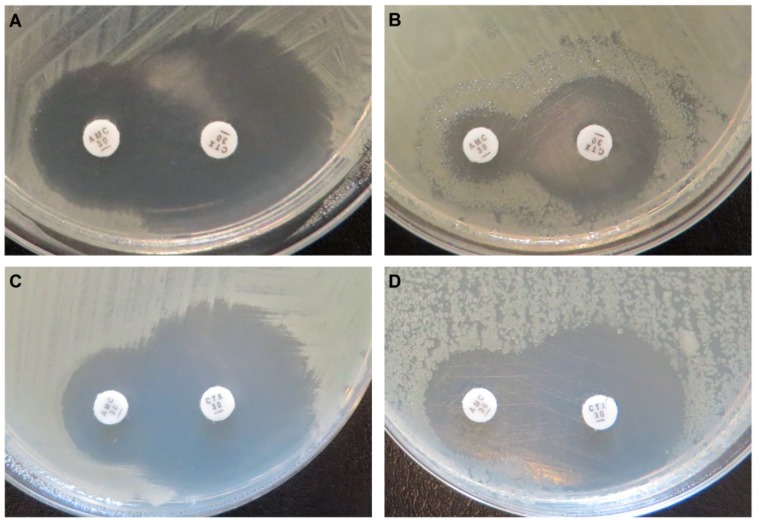
Disk diffusion tests. *E. coli* C600Z1 pZE21-*bla*_IDC-1_ with expression not induced (**A**) and induced (**B**), and *E. coli* C600Z1 pZE21-*bla*_IDC-2_ with expression not induced (**C**) and induced (**D**). AMC: amoxicillin + clavulanic acid; CTX: cefotaxime. The tests show that the activity of IDC-1 and IDC-2 is not inhibited by clavulanic acid.

Both novel AmpCs present the typical class C β-lactamase resistance profile (Table 1 and Table 2). Integron-derived cephalosporinase 1 (IDC-1) is active against penicillin, second- and third-generation cephalosporins (cefotaxime, ceftazidime, cefoxitin), and monobactams (aztreonam), but showed no clear activity against fourth-generation cephalosporins (cefepime) and carbapenems. In addition, it is unimpaired by clavulanic acid (Figure 2), but inhibited by cloxacillin [57,59,60]. The overexpression of *bla*_IDC-1_ caused a four-fold increase in the ertapenem MIC (Table 2). However, the strain remains susceptible (clinical breakpoint of Enterobacterales = 0.5 µg/mL; EUCAST breakpoint tables v9.0) under the tested conditions. The initial functional selections were carried out at concentrations designed to identify high-level ertapenem resistance, and were therefore too high to detect this small increase (0.032 µg/mL and 1 µg/mL, [48]). Expression of *bla*_IDC-2_ resulted in a generally minor increase of resistance against the same antibiotics, and was also not inhibited by clavulanate, but cloxacillin abolished the resistance phenotype. Decreased susceptibility to ceftazidime, amoxicillin, and aztreonam could not be revealed by disk diffusion tests (Table 1). When tested with Etest stripes, *bla*_IDC-2_ caused six-fold (aztreonam), four-fold (ceftazidime), and three-fold (amoxicillin) MIC increases, while it showed no activity against cefepime and carbapenems (Table 2). 

**Figure 3:**
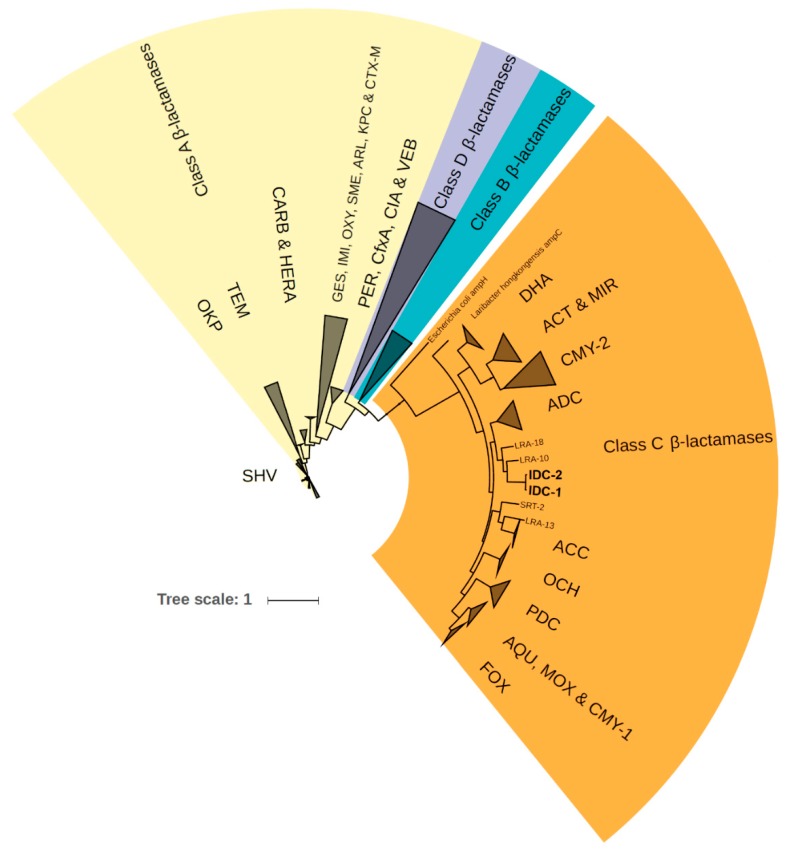
Phylogenetic tree of all four β-lactamase classes and the two newly discovered integron-derived AmpC variants IDC-1 and IDC-2.

Most known AmpCs have high isoelectric points [3,4], but the newly discovered AmpCs are predicted to have exceptionally low isoelectric point (pI) values (IDC-1: pI = 5.7, and IDC-2: pI = 5.1), indicating polarity changes compared to known AmpCs. Isoelectric focusing has been used to discern the different classes of β-lactamases, since the majority of AmpC proteins show considerably higher isoelectric points (pI ≥ 8) than class A β-lactamases [3,4,61,62]. The closely related AmpCs LRA-10 and LRA-18 (Figure 3) from the CARD database have predicted pI values of 9.4. Thus, the novel AmpC proteins cannot be distinguished from class A β-lactamases by isoelectric focusing. 

### 2.3. Phylogenetic Affiliation within Class C β-lactamases

The two novel AmpC variants named IDC-1 and IDC-2 formed a distinct cluster in the phylogenetic tree of β-lactamases from the CARD database (Figure 3), which led us to consider them as a new family of class C β-lactamases. The closest related proteins from the NCBI non-redundant protein database are WP_123086336.1 and WP_095207490.1, which are encoded by chromosomal genes in the Chinese soil isolate *Lysobacter* sp. ZS60 [63] and *Luteimonas* sp. JM171 from an Hawaiian coral [64]. These showed 85% and 72% amino acid identity to IDC-1, respectively, and have been annotated as class C β-lactamases, even though their functionalities are not proven. The closest homologous proteins with proven class C β-lactam resistance are LRA-10 and LRA-18. These proteins are encoded by genes recovered from Alaskan soil [65] (Figure 3) and share 55% identity with the two novel AmpCs. 

In order to detect the spread of the *bla*_IDC_ family, 1251 metagenomic datasets were searched for reads indicating their presence (Table S2 in the Supplementary Materials). The search revealed that reads mapping to *bla*_IDC_ are currently not common, but they appear in metagenomes prepared from wastewaters or river sediments contaminated with urban and pharmaceutical production wastewaters [53,66,67,68,69,70] especially in Asia (India, China, Vietnam, Cambodia). The gene seems to be still rare in the rest of the world. 

## 3. Discussion

Here we show an additional mode of *ampC* transmission. This is the first description of a class C β-lactamase, the *bla*_IDC_ family, expressed as gene cassettes under the control of a class 1 integron. The pool of gene cassettes, from which integrons can assemble their cassette array, contains class A, B, and D β-lactamases [42], and is now shown to be comprised of class C β-lactamases, too. The ability of integrons to acquire several resistance gene cassettes [36] provides ample opportunities for co-selection and further spread of a cassette. *AmpC* gene cassettes discovered in this study occur in combination with *bla*_GES-5_ or *bla*_OXA-10_, showing the potential for transmission of resistance genes against virtually all β-lactams in a single transfer event. Class 1 integrons are often further embedded into transposable elements and conjugative plasmids that have facilitated their spread into many versatile bacterial species thriving in various geographical and environmental conditions [71]. The *ampC* cassette is in all likelihood not limited to the contexts identified in this study, since integrons constantly acquire, excise, or shuffle their cassettes to adapt to changing conditions [72]. 

The two novel *ampC* gene cassettes are divergent from the known clinical *ampC* genes, but they are already mobilized and appear in an environment strongly impacted by hospital wastewater [53]. While not (yet) detected in the clinics, they occur in samples contaminated with municipal and hospital wastewater, indicating their potential to spread to human pathogens—or that they are already there but have escaped discovery, as was the case with the *gar* gene [48]. A systematic search for *bla*_IDC-1_ in available metagenomes proved that it is still rare and mainly present in Asian wastewaters (Table S2 in the Supplementary Materials). The integron-borne *ampC* genes were thus found in environments that both contain pathogens, which might already carry them, and may supply the selective forces necessary to drive their spread into and among human pathogens [73]. 

The most similar proteins currently available (January 2020) in the public databases show 85% or less identity to IDC-1. Hence, the origin of the novel AmpCs remains elusive. The novel class C β-lactamase genes were therefore named *bla*_IDC-1_ and *bla*_IDC-2_ (integron-derived cephalosporinase) upon consultation with the NCBI and in agreement with the current β-lactamase nomenclature. 

Penicillin-binding proteins and β-lactamases show very different binding affinities and hydrolyzing activities against various β-lactams, despite having similar structures and conserved motifs [3,74,75]. While both IDCs display a resistance pattern consistent with that of an AmpC, IDC-1 causes much higher resistance levels than IDC-2 in an isogenic background. All three conserved sequence motifs necessary for class C β-lactamase activity are identical in IDC-1 and IDC-2 [76,77]. However, 18 amino acid changes were detected outside of the conserved motifs, of which one or several might be responsible for the observed differences in activity. Modelling of the protein structures revealed that IDC-1 contains a positively charged Arg-238 close to the active site cavity, whereas IDC-2 harbors proline at this position (Figure S2, Supplementary Materials). Furthermore, only overexpression of IDC-1 caused a slight increase of resistance against ertapenem. This phenomenon has been previously reported in *Enterobacter cloacae*, *Citrobacter freundii*, and some plasmid-mediated AmpCs [27,78,79]. It points to an increased hydrolyzation activity towards carbapenems, but such a slight decrease in carbapenem susceptibility as caused by IDC-1 alone might not be of any clinical relevance. However, IDC-1 could very well (like other AmpCs) show enhanced resistance against an extended spectrum of β-lactams, including carbapenems, when occurring in a membrane porin-deficient background [6,79,80]. Several residues are involved in determining substrate affinity and hydrolyzation activity of class C β-lactamases [79,81]. Mutations in these positions could influence their ability to hydrolyze carbapenems. For example, Asn-346 is conserved in the AmpC proteins CMY-2, ACT-1, and DHA-1 that can confer carbapenem resistance, and was shown to be important for enhanced carbapenem hydrolysis, while Ile-346 occurs in AmpCs that fail to confer carbapenem resistance [82]. Both new AmpC proteins harbor Ile at the corresponding position (here: 374) (Figure S2, Supplementary Materials). The Ala-105 residue in some PDCs from *P. aeruginosa* also contributes to increased carbapenem resistance [83]. IDC-1 indeed harbors alanine at the corresponding position (here 109) and showed slightly reduced ertapenem susceptibility, in contrast to IDC-2 containing threonine at this position. Therefore, it is possible that the increased carbapenemase activity of IDC-1 is due to Ala-109, and might be further enhanced by a single point mutation at position 374. 

Since gene cassettes accumulate within an integron, the combination of *ampC* with other β-lactamase genes can lead to resistance against (almost) all β-lactams. In this study, we identified three different contexts of the *bla*_IDC-2_ gene, while the extended context of *bla*_IDC-1_ remained elusive (Figure 1). However, many more contexts are conceivable. GES-5 is an extended-spectrum class A β-lactamase active against penicillins, cephalosporins, cephamycins, and carbapenems; it is inhibited by clavulanate, sulbactam, and tazobactam, and is weakly/not active against ceftazidime and aztreonam [84]. These “gaps” in the resistance profile are covered by class C β-lactamases like IDC, which are not incapacitated by the mentioned inhibitors. OXA-10 enzymes (class D β-lactamases) generally show a narrow spectrum of hydrolysis, but some expanded spectrum variants are known [54,85]. The combination with an AmpC-type enzyme extends the resistance spectrum to all β-lactams except carbapenems. Single amino acid changes or a different genetic context can then complete the spectrum of activity [3,54]. Furthermore, class D β-lactamases hydrolyze cloxacillin [1], an inhibitor of class C enzymes, which leads to prolonged activity of AmpC during therapy.

Hyperproduction of AmpC due to one or several mutations/insertions or mobilization into a different context causes between eight-fold and up to 1000-fold increased expression [3,32,86,87,88,89]. These mechanisms lead to a resistance advantage offered by *ampC* expression prior to antibiotic exposure, but could be a greater fitness burden for the host in comparison to the more restricted chromosomal *ampC* expression. Gene cassettes, however, are expressed under the control of a constitutive promoter P_c_, which is specific for the gene cassette array [38]. The cassettes’ expression level is not just determined by the strength of the promoter, which can vary up to 30-fold [90,91], but also by the distance from the promoter [37], and can thus be changed by insertion or excision of other gene cassettes. The possible removal or reshuffling of *ampC* gene cassettes within the integron may provide a less costly control mechanism than the constant de-repression or even overexpression of many other (plasmid-borne) AmpCs. Moreover, gene cassettes could potentially be integrated into all integrons available within a bacterial genome, which would lead to an adaptable expression level. The interplay of many factors determines fitness and hampers our ability to judge if or how fast the integron-regulated *ampC* can spread. However, considering the enormous success of (mobile) integron-borne antibiotic resistances [41], this mode of *ampC* transmission has the potential to become more abundant. 

## 4. Materials and Methods

### 4.1. Detection of ampC Gene Cassettes

All complete and draft bacterial genomes in the NCBI assembly database (downloaded on 15 August 2019, containing 333,456 assembly reports) were searched for known *ampC* genes in gene cassettes. Reference *ampC* genes were identified by searching the AmpC serine hydrolase domain (conserved protein domain: PRK11289) against the CARD database [49] (v3.0.5) using Hmmer3.0 [92]. The search resulted in 311 proteins with e-values less than 10^−5^ (Supplementary File 2). By using Diamond [93] (v0.9.24.125), all genomes with at least one antibiotic resistance gene from the CARD database were extracted; all ORFs on the selected genomes were identified using Prodigal [94] (v2.6.3). Then, the predicted ORFs were searched against the reference AmpC dataset, using Diamond, with identity greater than 70% and coverage greater than 50% as thresholds. These thresholds were chosen to capture all known *ampC*s and allow for detection of new *ampC*s, while still limiting noise from penicillin binding proteins that do not confer β-lactam resistance. The DNA sequences ±2.5 kb up- and downstream of the 20,836 resulting ORFs were searched for integron attachment sites (*attC*) and the integrase gene using IntegronFinder [50]. 

In addition, the functional metagenomics dataset (NCBI BioProject database PRJNA555822) was searched for presence of *ampC* as gene cassettes. These amplicons contain gene cassettes amplified with primers specific for class 1 integrons, followed by functional selection for different antibiotic resistances [48]. We focused on the reads recovered from cefotaxime selection plates. First, the ORFs in the selected reads were identified using Prodigal, followed by a search against the reference AmpC dataset applying the aforementioned thresholds. To remove duplicates, the detected *ampC* were clustered with 100% identity using CD-HIT software [95,96]. To be considered an *ampC* gene cassette, the ORF needed to be complete and flanked by attachment sites. From the resulting 15 candidates, and according to the rationale described in the results section, two ORFs were chosen for synthesis, functional verification, and determination of a detailed resistance profile. Nucleotide sequences containing the novel *ampC* variants were deposited in GenBank (accession number MN985649: *bla*_IDC-1_; MN985646–MN985648: *bla*_IDC-2_ in different gene cassette arrangements; see Figure 1). 

### 4.2. Resistance Profile

The genes *bla*_IDC-1_ and *bla*_IDC-2_ were synthesized and subcloned into pZE21-MCS1 using KpnI and BamHI restriction sites by GeneArt Gene Synthesis (ThermoFisher Scientific, Regensburg, Germany), as described earlier [97]. The recombinant plasmids were electroporated into *E. coli* C600Z1 (Expressys, Bammental, Germany) and selected using the kanamycin resistance of pZE21-MCS1. The same strain with the empty vector was used as negative control. 

#### 4.2.1. Minimal Inhibitory Concentration Determination

Minimal inhibitory concentrations (MICs) were determined using Etest stripes (bioMérieux SA, Marcy l’Etoile, France) on MH (Mueller Hinton) agar plates supplemented with 50 µg/mL kanamycin, with the addition of 250 ng/ml anhydrotetracycline as an inducer of the P_LtetO-1_ promoter to ensure maximal expression of the *bla*_IDC_ genes. 

#### 4.2.2. Disk Diffusion Tests

Disk diffusion tests (Oxoid, Hampshire, United Kingdom) were performed according to EUCAST recommendations version 7.0 (2019). MH agar plates were supplemented with 50 µg/mL kanamycin, 250 ng/mL of the inducer anhydrotetracycline, and/or 50 µg/mL cloxacillin (class C β-lactamase inhibitor). Cultures were incubated at 37 °C for 24 h before measuring inhibition zones. Each disk of cefotaxime, ceftazidime, cefoxitin, cefepime, and aztreonam contained 30 µg of the antibiotic. Ertapenem disks contained 10 µg, and the amoxicillin–clavulanate combination disk contained 30 µg of the antibiotic and 10 µg of the inhibitor. Potential inhibition of the β-lactamase by clavulanic acid was controlled in a double disk synergy test by placing the cefotaxime and the amoxicillin–clavulanate combination disk at a distance of 20 mm center to center. 

### 4.3. Metagenome Search

The abundance of the *bla*_IDC_ family was searched in 1251 public metagenomics datasets, as described in [48]. The reads were mapped with Diamond [93] (v0.9.24.125) to the reference protein IDC-1, with thresholds of 95% identity and an ORF length greater than 20 amino acids to recover both *bla*_IDC-1_ and *bla*_IDC-2_ containing reads. Integron attachment sites were detected by mapping the short paired-end reads to the gene cassettes from Figure 1 using bowtie2 [98] (v2.2.9), and then manually analyzing the marginal paired-end reads using the Tablet software [99] (v1.19.05). 

### 4.4. Phylogenetic Tree

Amino acid sequences of *bla*_IDC-1_, *bla*_IDC-2_ and all available 1632 β-lactamases from CARD [49] (v3.0.5) were aligned using MAFFT [100] (v7.310; --maxiterate 1000 --localpair). The phylogenetic tree was calculated by FastTree [101] (v2.1.9) using the maximum likelihood algorithm, Jones–Taylor–Thornton model with 1000 times bootstrap. The full version of the tree is available in Supplementary File 3 (Newick format) and the proteins are listed in Supplementary File 4. The Interactive Tree Of Life (iTOL v4) online tool [102] (https://itol.embl.de; last access: January 2020) was used to prepare the phylogenetic tree for display. 

### 4.5. Amino Acid Sequence Analysis and Protein Model

The EMBOSS pepstats software (https://www.ebi.ac.uk/Tools/seqstats/emboss_pepstats/; last access: November 2019) was used to estimate the isoelectric point (pI) of the AmpC proteins. The signal peptide cleavage site was predicted by the SignalP 5.0 server [103]. The I-TASSER server for protein structure and function prediction was used to create models of the two AmpCs [104,105,106]. The models with the highest confidence scores (C-score IDC-1: 1.00 and IDC-2: 0.85) are shown in Figure S2. PyMOL v2.3.3 (https://pymol.org/2/; last access: January 2020) was used to create the ribbon presentation. 

## Figures and Tables

**Table 1:** Resistance profile of *bla*_IDC-1_ and *bla*_IDC-2_ (disk diffusion).

β-Lactam	Empty Vector				*bla*_IDC-1_				*bla*_IDC-2_			
			Cloxacillin				Cloxacillin				cloxacillin	
	Not Induced	Induced	Not Induced	Induced	Not Induced	Induced	Not Induced	Induced	Not Induced	Induced	Not Induced	Induced
Amoxicillin + clavulanate	21	22	n.d.	n.d.	24	13	n.d.	n.d.	21	20	n.d.	n.d.
Cefotaxime	35	35	36	37	38	24	38	36	35	32	35	43
Ceftazidime	32	31	32	33	32	27	34	35	31	32	32	41
Cefoxitin	27	29	31	31	28	17	32	29	28	25	31	29
Cefepime	33	33	34	34	36	37	36	37	33	43	35	44
Ertapenem	35	34	36	36	38	38	37	38	33	41	35	46
Aztreonam	32	32	35	35	36	30	37	37	33	36	34	43

Inhibition zone sizes are in millimeters; the increased resistance phenotype is underlined; n.d. = not determined. The expression of *bla*_IDC-1_ and *bla*_IDC-2_ was induced by supplementing the agar with anhydrotetracycline. Cloxacillin is an inhibitor of class C β-lactamases, and is shown to impair the resistance conferred by both IDCs.

**Table 2:** Resistance profile of *bla*_IDC-1_ and *bla*_IDC-2_ (Etest).

β-Lactam	Empty Vector			*bla*_IDC-1_			*bla*_IDC-2_		
	Not Induced	Induced	MIC Increase (Fold-Change)	Not Induced	Induced	MIC increase (Fold-Change)	Not Induced	Induced	MIC Increase (Fold-Change)
Amoxicillin	6	6	-	6	48	8	6	16	3
Cefotaxime	0.047	0.047	-	0.064	8	125	0.032	0.75	23
Ceftazidime	0.064	0.094	-	0.094	1.5	16	0.094	0.38	4
Cefoxitin	3	3	-	2	16	8	3	8	3
Cefepime	0.032	0.032	-	0.023	0.032	-	0.023	0.032	-
Ertapenem	0.003	0.003	-	0.004	0.016	4	0.003	0.004	-
Imipenem	0.50	0.50	-	0.38	0.50	-	0.38	0.38	-
Aztreonam	0.094	0.094	-	0.094	1.5	16	0.064	0.38	6

Minimal inhibitory concentration (MIC) values are in µg/ml. The expression of *bla*_IDC-1_ and *bla*_IDC-2_ was induced by supplementing the agar with anhydrotetracycline.The increased resistance phenotype is underlined.

## Supplementary Materials

The following are available online at https://www.mdpi.com/2079-6382/9/3/123/s1, Figure S1: Context and alignment of *bla*_CMY-2_ and an adjacent class 1 integron; Figure S2: Amino acid sequence and comparison of the predicted protein structures of IDC-1 and IDC-2; Table S1: Frequencies of unique PacBio long reads (97% identity threshold) that harbor novel *ampC* genes; Table S2: Occurrence of the newly discovered *bla*_IDC_ family in 1251 metagenomic datasets. Supplementary File 2: 311 AmpC proteins from CARD. Supplementary File 3: Phylogenetic tree of 1632 β-lactamases plus IDC-1 and IDC-2 in Newick format. Supplementary File 4: List of the 1632 β-lactamases from CARD.
