# Supplementary material for: A Novel, Integron-Regulated, Class C β-Lactamase
Source: Antibiotics (Basel). 2020 Mar 14;9(3):123. doi: 10.3390/antibiotics9030123 (PMC7148499; doi:10.3390/antibiotics9030123)
Supplement: Supplementary file 1 [file antibiotics-09-00123-s001.zip › antibiotics-731190-supple/antibiotics-731190-SUPPLE.pdf]

Supplement

Table S1. Frequencies of unique PacBio long reads (97 % identity threshold) that harbor novel *ampC* genes. Only reads containing ORFs with 100 % nucleotide identity and 100 % coverage to $bla_{\mathrm{IDC-1}}$ or $bla_{\mathrm{IDC-2}}$ are included, grouped by antibiotic selection sets. As expected, the ORF $bla_{\mathrm{IDC-2}}$ is most common in the cefotaxime selection set, but also occurs in the ertapenem and ciprofloxacin selection sets, while it does not confer resistance to these two antibiotics. A plausible explanation is that a $bla_{\mathrm{GES-5}}$ gene present on the same read provides resistance in all three appearances in the ertapenem selection set. The single read recovered from the ciprofloxacin selection set contains no factor which could explain the resistance phenotype. However, since the read is not complete (i.e. only one of the binding sites for the primers used to amplify the gene cassettes could be detected) another gene cassette might provide ciprofloxacin resistance. It is also conceivable that this clone was selected due to a chromosomal mutation conferring resistance to ciprofloxacin, for example in *gyrA*.

Selection set	Abundance of $bla_{\mathrm{IDC-1}}$	Abundance of $bla_{\mathrm{IDC-2}}$
**cefotaxime**	8	21
**chloramphenicol**	0	0
**ciprofloxacin**	0	1
**colistin**	0	0
**ertapenem**	0	3
**gentamicin**	0	0
**imipenem**	0	0
**meropenem**	0	0
**rifampicin**	0	0
**sulfamethoxazole**	0	0
**trimethoprim**	0	0

Table S2. Occurrence of the newly discovered *bla*$_{IDC}$ family in 1251 metagenomic datasets. The number of paired-end Illumina short reads mapping to *bla*$_{IDC-1}$ with 95 % identity over the length of at least 20 amino acids are shown. Using this threshold, both *bla*$_{IDC-1}$ and *bla*$_{IDC-2}$ containing reads are recovered. Reads from selected metagenomic datasets (111, 109, 105 from MG-RAST project: MGP19878) were mapped to DNA sequences containing *ampC* and surrounding attachment sites. Paired-end reads covered *ampC* and the adjacent attachment site, unambiguously confirming that *ampC* is present as a gene cassette. See [1] for the list of all metagenomes investigated.

Metagenome	Raw count	Environment	Country	Latitude	Longitude	Reference
111	135	Wastewater/sludge	India	19.0760	72.8777	[2]
109	56	River sediment *	India	18.5104	73.8399	[3]
105	38	River sediment *	India	18.5431	73.8916	[3]
104	37	River sediment *	India	18.5104	73.8399	[3]
110	27	River sediment *	India	18.5431	73.8916	[3]
103	17	River sediment *	India	18.4743	73.8091	[3]
ERR1713344	12	Wastewater/sludge	China	23.1114	113.352	[4]
JL32	10	Industrially polluted **	India	17.5407	78.2618	[5]
ERR1713407	9	Wastewater/sludge	Vietnam	10.8231	106.6297	[4]
ERR1713370	8	Wastewater/sludge	Cambodia	11.5449	104.8922	[4]
108	5	River sediment *	India	18.4743	73.8091	[3]
SRR2107218	5	Wastewater/sludge	Argentina	-34.07	-59.07	[6]
ERR1713410	4	Wastewater/sludge	Zambia	15.3875	28.3228	[4]
ERR2592258	3	Wastewater/sludge	India	9.9715	76.3051	[4]
ERR1713345	2	Wastewater/sludge	Cote d'Ivoire	5.3600	-4.0083	[4]
ERR1713355	2	Wastewater/sludge	Ethiopia	8.9199	38.7556	[4]
ERR1713362	2	Wastewater/sludge	India	9.9312	76.2673	[4]
ERR1713367	2	Wastewater/sludge	Italy	41.8193	12.4281	[4]
ERR1713369	2	Wastewater/sludge	Kenya	-1.2455	37.0161	[4]
ERR1713385	2	Wastewater/sludge	Peru	-11.997	-76.8368	[4]
ERR1713386	2	Wastewater/sludge	Senegal	14.7645	-17.3660	[4]
ERR1725951	2	Wastewater/sludge	Brazil	-1.4558	-48.4902	[4]
ERR1725985	2	Wastewater/sludge	Kenya	-1.2455	37.0161	[4]
ERR2592269	2	Wastewater/sludge	Singapore	1.3332	103.7561	[4]
ERR2592283	2	Wastewater/sludge	Zambia	-12.8232	28.2176	[4]
SRR2107176	2	Wastewater/sludge	Argentina	-34.07	-59.07	[4]
ERR1470826	1	Wastewater/sludge	Denmark	55.6950	12.6150	[4]
ERR1713337	1	Wastewater/sludge	Brazil	-1.4558	-48.4902	[4]
ERR1713368	1	Wastewater/sludge	Kazakhstan	43.4039	76.8892	[4]
ERR1713384	1	Wastewater/sludge	Pakistan	24.8615	67.0099	[4]
ERR1713387	1	Wastewater/sludge	Singapore	1.3521	103.8198	[4]
ERR1713405	1	Wastewater/sludge	USA	39.8714	-104.2701	[4]
ERR1726032	1	Wastewater/sludge	Zambia	-12.8232	28.2176	[4]
ERR1726033	1	Wastewater/sludge	Zambia	-12.8232	28.2176	[4]
ERR2592252	1	Wastewater/sludge	Cote d'Ivoire	5.3600	-4.0083	[4]
ERR2592255	1	Wastewater/sludge	Finland	60.2254	24.9940	[4]
ERR2592328	1	Wastewater/sludge	Australia	-35.3452	149.0950	[4]
JL28	1	Industrially polluted **	India	17.5513	78.2331	[5]
Metagenomics2	1	Industrially polluted **	India			[7]
SRR2107219	1	Wastewater/sludge	Argentina	-34.07	-59.07	[6]

* River sediment, contaminated with municipal and hospital wastewater

** River sediment, contaminated with wastewater from drug manufacture, including antibiotics

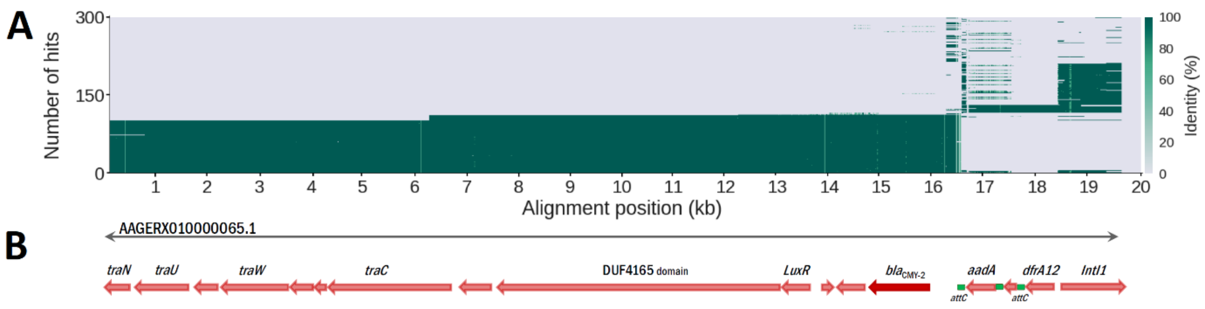

Figure S1. Context and alignment of *bla*$_{CMY-2}$ and an adjacent class 1 integron. **A:** Distribution of the conserved nucleotides across the reference sequence (AAGERX010000065.1). **B:** Annotation of the reference sequence consisting of the AmpC gene *bla*$_{CMY-2}$, a class 1 integron with two resistance gene cassettes (*aadA*, *dfrA12*) upstream and the *tra* conjugal transfer genes downstream. BLASTN results of the entire nucleotide sequence AAGERX010000065.1 against the GenBank database using online NCBI BLAST+ tools are shown. The *bla*$_{CMY-2}$ gene is part of a 16.5 kb region occurring on many plasmids, which lack the integron in their immediate upstream sequence. The location of the *attC* sites shows that *bla*$_{CMY-2}$ is not a gene cassette, but is simply located adjacent to the last cassette of the integron.

A

```
                                                  ↓
IDC-1      1  mprtesvpskslvvrtlllvfaclfpmavpavedssrvraavdaailplmsqhdipgmav
IDC-2      1  mprtesvpskslvvrtlllvfaclfpmavpavedtsrvrttvdaailplmsqhdipgmvv

IDC-1     61  glildgqpyvvtygvasketnvpvaeatlfeigsvskvftatlatyaqatgklslddhpg
IDC-2     61  glildgqpyvvtygvaskeanvpvaeatlfeigsvskvftatlaayaqttgklslddhpg

IDC-1    121  kylphlkgapidqatllhlgtytagglplqfpdevtgeaavmnyfrnwtplappgtrrey
IDC-2    121  kylpqlkgtpidqatllhlgtytagglplqfpdevtgevavmdyfrnwtplappgtrrey

IDC-1    181  snaspgllgvvaasaldddfatlmqttvfpafgmtdsfihvpdrkmpdyawgyrkdrrvr
IDC-2    181  snaspgllglvaasaldddfatlmqstvfpafgmtdsfihvpdrkmpdyawgyrkdrpvr

IDC-1    241  vnegpldeqaygvkttvsdllrfvqanidpnslepsmrhaveatqvgyfragtlvqqlgw
IDC-2    241  vnegpldeqaygvkttvsdllrfvqanidpsslepsmrraveatqvgyfragtlvqqlgw

IDC-1    301  ekypypvsrewllggnakemlfdpqpayrltdqtaggqylfnktgstggfatyvafvpar
IDC-2    301  ekypypvsrewllggnakemlfdpqpayrltdqtagerylfnktgstggfatyvafvpar

IDC-1    361  kigivmlanrsypipdrveaawmileqlasgtdsn  395
IDC-2    361  kigivmlanrsypipdrveaawiileqlasgtdsn  395
```

B

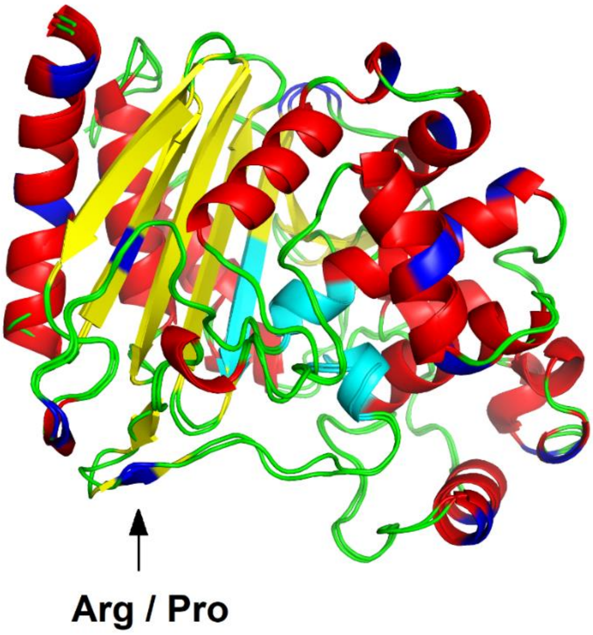

Figure S2. Amino acid sequence and comparison of the predicted protein structures of IDC-1 and IDC-2. While IDC-1 confers higher resistance than IDC-2, they have 95 % identity on amino acid level. One of the amino acid changes, Arg-238-Pro, is located close to the active site and might be responsible for the different level of β-lactamase activity. **A:** Alignment of IDC-1 and IDC-2 (precursor proteins). The four conserved sequence motifs are boxed in black [8,9]. Arg-238-Pro is boxed in red and the predicted signal peptide cleavage site for both proteins after position 31 is marked by an arrow. **B:** Overlay of the protein structure models IDC-1 and IDC-2 (mature proteins). Differences between the two AmpCs are highlighted in blue, the conserved active site in cyan.

References

1. Böhm, M.-E.; Razavi, M.; Marathe, N.P.; Flach, C.-F.; Larsson, D.G.J. Discovery of a novel integron-borne aminoglycoside resistance gene present in clinical pathogens by screening environmental bacterial communities. *Microbiome* **2020**, *In press.*
2. Marathe, N.P.; Berglund, F.; Razavi, M.; Pal, C.; Dröge, J.; Samant, S.; Kristiansson, E.; Larsson, D.G.J. Sewage effluent from an Indian hospital harbors novel carbapenemases and integron-borne antibiotic resistance genes. *Microbiome* **2019**, *7*, 97, doi:10.1186/s40168-019-0710-x.
3. Marathe, N.P.; Pal, C.; Gaikwad, S.S.; Jonsson, V.; Kristiansson, E.; Larsson, D.G.J. Untreated urban waste contaminates Indian river sediments with resistance genes to last resort antibiotics. *Water Res.* **2017**, *124*, 388-397, doi:10.1016/j.watres.2017.07.060.
4. Hendriksen, R.S.; Munk, P.; Njage, P.; van Bunnik, B.; McNally, L.; Lukjancenko, O.; Röder, T.; Nieuwenhuijse, D.; Pedersen, S.K.; Kjeldgaard, J., et al. Global monitoring of antimicrobial resistance based on metagenomics analyses of urban sewage. *Nat Commun* **2019**, *10*, 1124, doi:10.1038/s41467-019-08853-3.
5. Kristiansson, E.; Fick, J.; Janzon, A.; Grabic, R.; Rutgersson, C.; Weijdegård, B.; Söderström, H.; Larsson, D.G.J. Pyrosequencing of antibiotic-contaminated river sediments reveals high levels of resistance and gene transfer elements. *PLoS ONE* **2011**, *6*, e17038, doi:10.1371/journal.pone.0017038.
6. Ibarbalz, F.M.; Orellana, E.; Figuerola, E.L.M.; Erijman, L. Shotgun Metagenomic Profiles Have a High Capacity To Discriminate Samples of Activated Sludge According to Wastewater Type. *Appl. Environ. Microbiol.* **2016**, *82*, 5186-5196, doi:10.1128/AEM.00916-16.
7. Pal, C.; Bengtsson-Palme, J.; Kristiansson, E.; Larsson, D.G.J. The structure and diversity of human, animal and environmental resistomes. *Microbiome* **2016**, *4*, 54, doi:10.1186/s40168-016-0199-5.
8. Feller, G.; Zekhnini, Z.; Lamotte-Brasseur, J.; Gerday, C. Enzymes from cold-adapted microorganisms. The class C β-lactamase from the antarctic psychrophile *Psychrobacter immobilis* A5. *Eur. J. Biochem.* **1997**, *244*, 186-191, doi:10.1111/j.1432-1033.1997.00186.x.
9. Pozzi, C.; Di Pisa, F.; De Luca, F.; Benvenuti, M.; Docquier, J.D.; Mangani, S. Atomic-Resolution Structure of a Class C β-Lactamase and Its Complex with Avibactam. *ChemMedChem* **2018**, *13*, 1437-1446, doi:10.1002/cmdc.201800213.
